# Co‐evolution within the plant holobiont drives host performance

**DOI:** 10.15252/embr.202357455

**Published:** 2023-07-20

**Authors:** Fantin Mesny, Stéphane Hacquard, Bart PHJ Thomma

**Affiliations:** ^1^ Institute for Plant Sciences University of Cologne Cologne Germany; ^2^ Department of Plant Microbe Interactions Max Planck Institute for Plant Breeding Research Cologne Germany; ^3^ Cluster of Excellence on Plant Sciences (CEPLAS) Cologne Germany

**Keywords:** ecosystem, fitness, immunity, metaorganism, microbiome, Evolution & Ecology, Microbiology, Virology & Host Pathogen Interaction, Plant Biology

## Abstract

Plants interact with a diversity of microorganisms that influence their growth and resilience, and they can therefore be considered as ecological entities, namely “plant holobionts,” rather than as singular organisms. In a plant holobiont, the assembly of above‐ and belowground microbiota is ruled by host, microbial, and environmental factors. Upon microorganism perception, plants activate immune signaling resulting in the secretion of factors that modulate microbiota composition. Additionally, metabolic interdependencies and antagonism between microbes are driving forces for community assemblies. We argue that complex plant–microbe and intermicrobial interactions have been selected for during evolution and may promote the survival and fitness of plants and their associated microorganisms as holobionts. As part of this process, plants evolved metabolite‐mediated strategies to selectively recruit beneficial microorganisms in their microbiota. Some of these microbiota members show host‐adaptation, from which mutualism may rapidly arise. In the holobiont, microbiota members also co‐evolved antagonistic activities that restrict proliferation of microbes with high pathogenic potential and can therefore prevent disease development. Co‐evolution within holobionts thus ultimately drives plant performance.

## Graphical abstract

This review discusses how evolution of plant–microbe and intermicrobial interactions within plant holobionts impacts the performance of plants and their associated microorganisms.

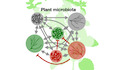

## Introduction

In nature, a wide diversity of microorganisms inhabit above‐ and belowground plant tissues (Müller *et al*, 2016; Gross, 2022). These communities are referred to as “plant microbiota” and comprise prokaryotes (i.e., bacteria and archaea) and eukaryotes (e.g., fungi, protists including oomycetes, algae, and even animals such as nematodes and small insects) that mostly rely on plant‐derived carbon compounds for energy production. By promoting plant growth, health, and resilience, microbiota generally are beneficial for their hosts (Durán *et al*, 2018; Gross, 2022). Accordingly, plants growing in the absence of microbiota are particularly vulnerable to disease and are unlikely to survive in natural settings (preprint: Paasch *et al*, 2023).

While a large fraction of the plant microbiota is horizontally acquired from the environment, some taxa are stably associated with their host, independently from soil and environmental conditions (Compant *et al*, 2019). A subset of these core microbiota members can be vertically transmitted through seeds, but also during clonal reproduction of the plant (Vannier *et al*, 2018; Abdelfattah *et al*, 2023). As they engage in stable associations, microbiota are likely to co‐evolve with their hosts. Accordingly, the phylogenetic relatedness of two plants typically correlates with the similarities of their microbial communities (Bouffaud *et al*, 2014; Kembel *et al*, 2014; Abdelfattah *et al*, 2022).

Since its inception, plant evolution is believed to be driven by interactions with microbes (Ramanan *et al*, 2016; Delaux & Schornack, 2021). Symbiotic associations with fungi are ancient and have been instrumental for land plant evolution, by facilitating the uptake of nutrients from soils and possibly even contributing to early root development (Kenrick & Strullu‐Derrien, 2014; Martin *et al*, 2017). Moreover, the chloroplasts and mitochondria that have become responsible for photosynthesis and aerobic respiration, respectively, are derived from ancient bacterial endosymbionts (Archibald, 2015). Furthermore, plant immune systems evolved under the continuous threat of pathogenic microbes (Cook *et al*, 2015; Delaux & Schornack, 2021).

Given their long‐lasting and continuous association with co‐evolving microbiota, plants may be considered as ecological entities, generally termed “holobionts,” rather than as singular organisms. Contrary to the definition of “meta‐organism” that implies specificity of time and place and a function for each member, a holobiont represents a eukaryotic host with all external and internal associates, regardless of their stability and functionality (Jaspers *et al*, 2019). A contrasting and more specific definition of the “plant holobiont” implies evolutionary selection between plants and microorganisms, contributing to an overall stability of the system (Trivedi *et al*, 2020; Lyu *et al*, 2021; Berg *et al*, 2023). Since stable associations concern only a fraction of the interactions in the plant holobiont, we adopt the former holobiont definition and consider that co‐evolution occurs within plant holobionts, although it may not involve all members. Arguably, plant evolution in the holobiont favors associations with beneficial partners and counter‐selects for detrimental interactions.

Experimental dissection of root‐ and leaf‐associated microbial communities revealed that the effects of microbiota members on host performance are diverse (Durán *et al*, 2018; Eitzen *et al*, 2021; Mesny *et al*, 2021). In addition to mutualists and commensals, both opportunistic and obligate pathogens may occur in holobionts (Box 1). Co‐evolution between plants and various types of pathogens with diverse lifestyles has been well‐documented and is often referred to as an “arms‐race” in which the plant evolves mechanisms to detect and intercept individual pathogens, while pathogens evolve to evade or suppress this interception to continue host colonization (Cook *et al*, 2015; Möller & Stukenbrock, 2017; Han, 2019). However, little is known about the evolution of nonpathogenic microbes in plant‐associated environments.

Box 1 **Pathogenic potential in plant microbiota**

Microbiota of healthy plants generally comprise microbes with disease‐causing potential (Cregger *et al*, 2018; Nelson & Shaw, 2019; Thiergart *et al*, 2020; Rudgers *et al*, 2022), which is evident from the so‐called “recolonization experiments” where isolates negatively affect plant health when individually inoculated on otherwise sterile plants (Kia *et al*, 2017; Nelson *et al*, 2018; Mesny *et al*, 2021). In holobionts, disease is mitigated by the combined action of plant immune systems and the collective microbiota composition that keeps microbes with pathogenic potential in check (Wolinska *et al*, 2021). In agreement with the “disease triangle” concept, stating that disease develops from the interplay of host genetics, pathogen genetics, and environmental factors, which can be understood to comprise microbiota composition besides other biotic and abiotic cues (McNew, 1960), disease development may be promoted by external stimuli that increase stochasticity in plant microbiota assemblies and therefore affect mitigative forces (Arnault *et al*, 2023).

Given that microbes with disease‐causing potential frequently occur in healthy holobionts, we should consider how to treat pathogens. However, rather than classifying microorganisms as commensals, pathogens, or mutualists with strictly separated lifestyles, we need to realize that this classification occurs as a continuum. Accordingly, the ability to cause disease (i.e., pathogenicity) should be appreciated as a continuous variable (i.e., the “pathogenic potential”) incorporating the occurrence of host damage, time, and virulence factors (Casadevall, 2017, 2022). Microorganisms with low pathogenic potential may not (or rarely) cause disease, presently often referred to as opportunistic pathogens, while the ones with the highest pathogenic potential are virulent, obligate pathogens. The development of disease may be determined by the extent of microbial colonization, which depends on the holobiont composition, which in turn is modulated by environmental stimuli. Thus, importantly, pathogens are part of holobionts too, irrespective of their infection strategy. This concept agrees with the endophytic continuum theory, stating that the interaction of fungal endophytes with their hosts is never neutral (Schulz & Boyle, 2005). A particular degree of “virulence” is required to enable host colonization of plant tissues, and disease establishment is eventually repressed by plant immunity and environmental factors.

When immunity is disrupted, or environmental factors are permissive, plants may develop disease depending on the pathogenic potential of their microbiota members (Plett *et al*, 2014; Lahrmann *et al*, 2015; Hiruma *et al*, 2016; Chen *et al*, 2020a; Wolinska *et al*, 2021). Accordingly, defense responses targeting all plant‐colonizing microorganisms, including those with performance‐promoting effects, are essential for plant survival and fitness. The common conception that plants evolved immune systems to defend against particular “pathogens” is thus incongruous, and immunity should be considered as an organism's capacity to withstand any microbial invader that may cause damage, which can be any member of their microbiota.

Complex plant–microbe and intermicrobial interactions, together with environmental cues, influence microbiota structure and composition (Müller *et al*, 2016; Hassani *et al*, 2018). Likely, many mechanisms behind these interactions result from co‐evolution within the holobiont. In this review, we assemble evidence that supports co‐adaptation within the holobiont as a driver for plant performance.

## Plant immune systems shape beneficial microbiota composition

While plants evolved continuously surrounded by a wealth of microorganisms, they established diverse symbiotic associations ranging from detrimental to beneficial (Han, 2019). Simultaneously, they evolved a complex innate immune system. Pathogen‐induced biotic stresses affecting fitness have long been considered the sole drivers of plant immune system evolution. However, numerous studies have demonstrated that also nonpathogenic microbes are recipients of plant immune responses, and they likely contributed to shaping them (Teixeira *et al*, 2019; Yu *et al*, 2019a; Box 1). An increasingly accepted view proposes that immune systems act as microbial management systems to control microbiota assembly and host–microbe homeostasis (Cook *et al*, 2015; Hacquard *et al*, 2017; Teixeira *et al*, 2019). After introducing how plant immune systems operate, we will show that plant microbiota are shaped by immune responses and argue that plants evolved mechanisms to mediate beneficial microbiota assembly.

### Plant immune systems are integrated surveillance systems

While recognized as integrated surveillance systems to detect microbial invasion (Thomma *et al*, 2011; Cook *et al*, 2015), plant immune systems have been molecularly portrayed as composed of two layers (Chisholm *et al*, 2006; Jones & Dangl, 2006), recently realized to be mechanistically strongly interconnected (Pruitt *et al*, 2021; Parker *et al*, 2022; Feehan *et al*, 2023). As a first layer, plasma membrane‐localized receptors known as pattern recognition receptors (PRRs) perceive conserved structural molecules, such as bacterial lipopolysaccharides and fungal chitin, referred to as microbe‐associated molecular patterns (MAMPs). These receptors are known as pattern recognition receptors (PRRs), and they include receptor‐like kinases (RLKs) and receptor‐like proteins (RLPs). Recognition of MAMPs leads to the activation of immune signaling to establish MAMP‐triggered immunity (MTI). Major immune signaling pathways are mediated by reactive oxygen species (ROS) and by the phytohormones salicylic acid (SA), jasmonic acid (JA), and ethylene (ET) (Peng *et al*, 2018). Local concentrations in these compounds change upon MAMP perception and mediate activation of plant defenses. Analysis of transcriptomic reprogramming upon perception of different MAMPs in *Arabidopsis thaliana* identified a congruent set of rapidly activated responses that are also induced when plants face abiotic stresses, suggesting that early transcriptional responses to MAMPs overlap with general stress responses (Bjornson *et al*, 2021).

To overcome this first defense layer, microbes secrete small proteins, typically referred to as “effectors,” supporting host colonization by repressing or evading MTI, leading to “effector‐triggered susceptibility” (ETS) (Chisholm *et al*, 2006; Jones & Dangl, 2006). However, to counteract this breach of MTI, plants re‐activate immunity upon sensing the presence or activity of microbial effectors. This so‐called “effector‐triggered immunity” (ETI) was proposed to rely on intracellularly located receptors from the nucleotide‐binding domain leucine‐rich repeat containing (NLR) family. Like microbial effector catalogs, NLR gene repertoires are highly plastic and diversify rapidly, driven by duplication, translocation, deletion, and promiscuous gene exchange events (Han, 2019; van de Weyer *et al*, 2019). A recent genomic analysis showed that NLRs display higher variability than PRRs in *A. thaliana* (Pruitt *et al*, 2021). The perpetual adaptation of NLR sequences to rapidly evolving effector proteins, and of PRRs to MAMPs, is generally referred to as an “arms race” in which plants and microorganisms aim to detect and overcome detection, respectively. While ETI may culminate in host localized cell death, it also reinstates and amplifies MTI‐related defenses that were perturbed through microbial effector activities (Parker *et al*, 2022).

### Plant immune systems shape microbiota assemblies

Plant tissues are colonized by phylogenetically diverse microorganisms that collectively evolved a broad variety of MAMPs and effectors and may therefore differentially interact with plant immune systems. The impact of MTI responses on microbiota structure and composition is increasingly recognized. When treated with the bacterial flagellin‐derived MAMP epitope flg22, *A. thaliana* plants overexpressing the PRR‐encoding gene FLS2 revealed an effect of MTI responses on root microbiota composition (Ma *et al*, 2021). Consistently, the *A. thaliana* WRKY33 transcription factor, which positively regulates expression of immunity genes upon flg22 treatment, shapes root‐associated microbiota (Wolinska *et al*, 2021). In *A. thaliana*, a PRR complex that includes the receptor kinase FERONIA restricts rhizosphere colonization by *Pseudomonas* bacteria (Song *et al*, 2021). Upon phosphate starvation, PRR complex formation is repressed, leading to enrichment in the root microbiota of several bacterial genera that alleviate low‐phosphate stress (Tang *et al*, 2022). Deletion of three *A. thaliana* PRR genes (namely FLS2, EFR, and CERK1) together with a gene involved in vesicle‐trafficking (MIN7) resulted in an endophytic phyllosphere community shift and microbial overproliferation under high humidity, causing detrimental effects on plant performance (Chen *et al*, 2020a). Interestingly, deletion of the *A. thaliana* NADPH oxidase RBOHD, responsible for respiratory bursts that limit microbial colonization, has a greater impact on the leaf microbiota than deletion of PRR genes (Pfeilmeier *et al*, 2021). Taken together, these findings highlight the importance of MTI for microbiota modulation and the restriction of microbial proliferation in plant tissues. While the impact of ETI on microbiota assembly remains to be demonstrated, recent studies linking natural genetic variation in barley and sorghum to their rhizosphere microbiota revealed correlations between community composition and the presence of particular NLR genes (Deng *et al*, 2021; Escudero‐Martinez *et al*, 2022).

Although immune systems shape whole microbiota, mechanisms accommodating beneficial microbes while keeping pathogenic invaders at bay have been positively selected during plant evolution. This implies that plants can distinguish beneficial from detrimental microbes. This is the case in arbuscular mycorrhizal symbioses, in which plant RLKs perceive fungal short‐chain chitooligosaccharides and nonsulfated lipochitooligosaccharides, commonly referred to as “Myc factors,” resulting in the activation of symbiosis signaling and in the suppression of immunity to accommodate the mycorrhizal fungus (Feng *et al*, 2019). Similarly, to induce root nodule symbioses, rhizobia synthesize lipochitooligosaccharides after sensing the presence of legume‐secreted flavonoids. Upon perception of these lipochitooligosaccharides by host cell surface receptors, symbiotic programs are induced and immune signaling is repressed (Ghantasala & Roy Choudhury, 2022). Opportunities for mutualist associations to develop arise from the rapid evolution and high variability of MTI. Accordingly, intraspecific variation of immune responses upon MAMP perception occurs in *A. thaliana* (Vetter *et al*, 2016) and in tomato (Veluchamy *et al*, 2014; Roberts *et al*, 2019), while microorganisms may exhibit considerable variation in their MAMPs (Teixeira *et al*, 2019).

### Phytohormone signaling modulates plant microbiota composition

Downstream of MAMP and effector perception, the phytohormones salicylic acid (SA), and jasmonic acid (JA) mediate immune signaling as part of MTI and ETI (Venugopal *et al*, 2009; Liu *et al*, 2016; Cui *et al*, 2017). These two phytohormones were the first immunity components proven to directly impact microbiota diversity and composition. Induction of SA‐mediated defenses reduced endophytic bacterial community diversity in *A. thaliana* leaves, whereas plants deficient in JA‐mediated defenses revealed greater epiphytic diversity (Kniskern *et al*, 2007). In roots, both SA and JA influence bacterial community composition in the rhizosphere and in the endosphere (Lebeis *et al*, 2015). A third phytohormone of importance for plant immunity is ethylene (ET), a volatile compound produced at early MTI stages that transcriptionally activates FLS2 production in *A. thaliana* (Mersmann *et al*, 2010; Jones *et al*, 2019). Both the phyllosphere and the rhizosphere of ET‐insensitive *ein2* mutants host bacterial communities that significantly differ from wild‐type plants (Doornbos *et al*, 2011; Bodenhausen *et al*, 2014). Finally, brassinosteroids, phytohormones that act at the growth‐defense interface, indirectly shape root microbiota composition. In *A. thaliana*, these hormones bind to a molecular complex that includes the transmembrane receptor kinase BRI1. While this interaction activates growth, it also represses defense‐gene expression, callose deposition, ROS accumulation, and spontaneous cell death (He *et al*, 2007). Bacterial and fungal community profiling of BRI1 knock‐out plants revealed major differences when compared with wild‐type plants (Hou *et al*, 2021; Wolinska *et al*, 2021).

Fine modulation of phytohormone‐signaling mediates the accommodation of beneficial microbes in a context‐dependent manner. Treatment of bulk soil with methyl‐JA lowered the variability of rhizosphere microbial communities (Carvalhais *et al*, 2013). The JA‐induced community shift was associated with an enrichment of bacterial taxa known to suppress plant pathogens and herbivore attack, and with a depletion of growth‐promoting taxa. In the ectomycorrhizal association of *Laccaria bicolor* with *Populus trichocarpa*, JA signaling prevents intercellular fungal overcolonization to sustain a long‐term symbiotic association (Plett *et al*, 2014). Interestingly, foliar application of methyl‐JA on *Lotus japonicus* plants inhibited root nodulation, pointing to a potential role of JA signaling in the repression of rhizobial recruitment (Nakagawa & Kawaguchi, 2006). Therefore, plants may modulate JA signaling to remodel their microbiota in a context‐dependent manner, leading to the recruitment of microbes that can alleviate stress. Signaling mediated by other phytohormones also contributes to recruitment of beneficial microbiota members. For instance, ET is enriched in peanut roots grown in a cyanide‐rich soil. Cyanide triggered increased belowground production of ET, resulting in altered root microbiota composition and enrichment of *Catenulispora* bacteria (Chen *et al*, 2020b). The reshaped root microbiota correlated with increased soil ammonium, nitrogen, and available phosphorus concentrations, pointing to a role of ET in the assembly of beneficial microbiota to alleviate mineral stress. Thus, phytohormones are key mediators of plant microbiota composition.

### Immune‐related metabolites shape beneficial plant microbiota

Phytohormone signaling results in the production and secretion of compounds with direct effects on plant‐associated microorganisms. For instance, application of exogenous JA to *A. thaliana* roots alters root exudate profiles, correlating with changes in bacterial and archaeal communities in the rhizosphere (Carvalhais *et al*, 2015). While such exudates include hydrocarbon compounds that can be used by microbes as energy source, others, especially nucleotides, can act as chemotaxis signals to recruit microbes into the microbiota (Yang *et al*, 2015; Jones *et al*, 2019). Tryptophan‐derived metabolites, secreted as part of plant immune responses, act in shaping plant microbiota composition. *Arabidopsis thaliana* *cyp79b2‐cyp79b3* mutants do not convert tryptophan into indole‐3‐acetaldoxime, a precursor of indole glucosinolates, but also of camalexin and indole‐3‐carboxylic acids, and they host highly different root‐associated bacterial communities from wild‐type plants (Wolinska *et al*, 2021). These mutants are adversely affected by a synthetic microbial community that represents a native root microbiota, and by the beneficial fungi *Colletotrichum tofieldiae*, *Serendipita vermifera*, and *Serendipita indica* due to uncontrolled fungal growth in root endophytic compartments (Nongbri *et al*, 2012; Lahrmann *et al*, 2015; Hiruma *et al*, 2016; Wolinska *et al*, 2021). In other plants, tryptophan‐derived metabolites impact root‐associated microbial communities as well. Maize mutants that lack benzoxazinoids combine different root metabolomic profiles with altered bacterial community composition (Cotton *et al*, 2019; Cadot *et al*, 2021). Furthermore, root‐secreted antimicrobial triterpenes shape bacterial community composition in *A. thaliana* and melon (Huang *et al*, 2019; Zhong *et al*, 2022).

Plant metabolites and exudates are also used to actively recruit beneficial microbiota members through chemotaxis or growth stimulation (Sasse *et al*, 2018). For instance, *Pseudomonas putida* KT2440, a beneficial bacterium in the maize rhizosphere, is chemotaxically attracted to a benzoxazinoid in maize root exudates (Neal *et al*, 2012). Triterpenes act in the active selection of microbes through plant defense signaling and antimicrobial activities. *Arabidopsis thaliana* triterpene biosynthesis affects root microbiota composition, and purified triterpene cocktails differentially affect microbial growth (Huang *et al*, 2019). Consistently, cucurbitacins (bitter triterpenes) modulate rhizosphere composition of cucurbit plants, recruiting *Enterobacter* and *Bacillus* bacteria that protect against the soil‐borne fungal pathogen *Fusarium oxysporum* (Zhong *et al*, 2022). When colonized by the pathogen *Fusarium culmorum*, *Carex arenaria* roots emit a specific blend of volatile organic compounds (VOCs) that attract bacteria with antifungal properties (Schulz‐Bohm *et al*, 2018). Involved in iron nutrition, coumarins are semiochemical phenolic secondary metabolites that shape *A. thaliana* root microbiota (Stringlis *et al*, 2018a; Stringlis *et al*, 2019; Voges *et al*, 2019). Under iron limitation, *A. thaliana* reshapes its root microbiota by secreting coumarins to recruit beneficial microbes that alleviate iron stress (Harbort *et al*, 2020). Taken together, these findings illustrate that immune‐related metabolites shape plant microbiota and help plants to overcome various stresses.

## Microbiota evolved to persist without adverse effects on host plants

As plants evolved immune systems to face biotic stresses and shape their microbiota, microorganisms evidently adapted to plant‐associated environments. Microorganisms have evolved a plethora of mechanisms that promote host performance (Box 2). We will first detail known evolutionary trajectories of microbiota members. Then, we will detail how microbes adapt to plant immune responses. Next, we will argue that assimilation or deconstruction of plant carbohydrates is a major driver for microbial adaptation to plants. Finally, we will show that microbes can rapidly evolve to engage in mutualistic plant associations.

Box 2 **Mechanisms of plant growth promotion by microbiota members**

Microorganisms evolved a plethora of mechanisms to promote plant host performance. First, they provide nutrition. Root nodule‐forming rhizobia catalyze the reduction and ionization of atmospheric dinitrogen into ammonium to improve nitrogen acquisition by legumes (Singh *et al*, 2022). Other microbiota members mediate nutrient uptake from soil. The hyphae of mycorrhizal fungi take up mineral phosphate, ammonium, nitrate, sulphate, potassium, and water, and they provide them to the plant in exchange for carbohydrates and lipids (Martin *et al*, 2016; Liu *et al*, 2020; Berger & Gutjahr, 2021; Kakouridis *et al*, 2022). Rhizosphere microbiota likely act in host nutrition by processing nutrients to permit plant uptake. Many microbes can solubilize inorganic phosphate (Hiruma *et al*, 2016; Rawat *et al*, 2021). Moreover, microbes can relieve iron starvation by reducing ferric into ferrous ions that can be taken up by plant roots (Harbort *et al*, 2020). Likewise, other essential soil macro‐ and micronutrients can be solubilized, including potassium, zinc, and manganese (Singh *et al*, 2022).

Plant growth can also be promoted by microbes through interference with host metabolism, especially through modulation of phytohormone signaling. Microbial auxin production is a major plant growth‐promoting trait, since the most abundant auxin, indole‐3‐acetic acid, is synthesized by a broad variety of microbes (Bulgarelli *et al*, 2013). By activating root proliferation, auxin may increase absorption of nutrients and water. In contrast, ethylene inhibits root growth and, consequently, plant growth. Thus, some microorganisms secrete 1‐aminocyclopropane‐1‐carboxylate deaminase to convert an ethylene precursor to α‐ketobutyrate and ammonia, to prevent ethylene accumulation. Microbes may also promote growth through emission of volatile organic compounds, some of which modulate phytohormone signaling, while others promote photosynthesis (Tyagi *et al*, 2018).

In addition to these direct effects on plant growth, microbes may promote plant performance under abiotic and biotic stresses. Some protect hosts from environmental stressors that include light deprivation, salt stress, warming, heavy metal and cyanide pollution (Chen *et al*, 2020b; Hou *et al*, 2021; Carrell *et al*, 2022; Haque *et al*, 2022; King *et al*, 2022). Other microbes may antagonize potentially pathogenic microbes or elicit defense responses (Vogel *et al*, 2021) to prevent disease development in the holobiont (see subsection “Plant microbiota mediate disease suppression”).

### Microbiota members evolved from ancestors with diverse lifestyles

Microorganisms have interacted with land plants for 450 million years from their evolutionary origin onwards, and presently engage in a broad range of associations spanning the mutualism‐to‐parasitism continuum (Delaux & Schornack, 2021). However, the evolutionary trajectory of microbiota members remains poorly understood. Likely, plant‐adapted microbes originate from ancestors with diverse lifestyles. The bacterial capacity to fix nitrogen evolved several times independently, in association with plants that are genetically predisposed to form root nodules (Martin *et al*, 2017). Given the differences in root colonization strategies, nitrogen‐fixing bacteria likely derive from ancestors with different lifestyles. The adaptation of mycorrhizal fungi to plant roots results from long‐term genome evolution, involving extensive gene losses. Glomeromycotina arbuscular mycorrhizal fungi possibly evolved from ancestors forming endosymbioses with cyanobacteria, and the emergence of mycorrhiza was concomitant with substantial gene losses in thiamine sugar and fatty acid metabolism (Mahdi *et al*, 2021). Ectomycorrhizal fungi from the Agaricomycetes class are derived from saprotrophic ancestors by major losses of plant cell wall‐degrading enzymes (Kohler *et al*, 2015; Miyauchi *et al*, 2020). Some ancestral saprotrophs of the Agaricomycetes class evolved into endophytic fungi (Garnica *et al*, 2016; Mesny *et al*, 2021). However, most fungal endophytes in the *A. thaliana* root microbiota belong to the Sordariomycetes and Dothideomycetes, and they likely evolved from pathogenic ancestors (Mesny *et al*, 2021). It has been proposed that some endophytic fungi are “in the wait” for co‐evolution with their host and development of mycorrhizal symbiosis (Selosse *et al*, 2009, 2022). However, microbiota members can also evolve into virulent pathogens (Bhunjun *et al*, 2023). In both prokaryotes and eukaryotes, only few genes or transcriptomic changes can mediate the differentiation between beneficial and detrimental strains (Mohr *et al*, 2008; Hacquard *et al*, 2016; Melnyk *et al*, 2019; preprint: Hiruma *et al*, 2022; preprint: Thoms *et al*, 2023). Thus, gains and losses of pathogenicity frequently occur.

In advanced stages of their evolutionary history, some microbiota members adapted to particular host plants only. Endophytic shoot microbiota composition was correlated with host evolutionary distance in a study of 11 *Malus* species (Abdelfattah *et al*, 2022). The microbiota of domesticated apple tree (*Malus domestica*) is an admixture of its wild progenitors, suggesting introgression of microbial communities and supporting host‐microbiota co‐evolution during plant domestication. After bacterial isolation from the roots of *A. thaliana* and *L. japonicus*, reciprocal inoculations of sterile host plants with synthetic communities revealed a competitive advantage of native strains when colonizing roots of their cognate host, but not its rhizosphere (Wippel *et al*, 2021). As this host preference could neither be linked to plant immunity nor to root exudates, its genetic and evolutionary basis remains poorly understood. Host and nonhost communities did not show significant impact on plant performance but may help their host resist biotic and abiotic stresses in nature. Adaptation to specific plant species was also described for eukaryotic microbiota members, such as for the beneficial fungal endophyte *Epichloë typhina* (Schirrmann & Leuchtmann, 2015), which is reflected in their effector catalogs (Schirrmann *et al*, 2018). Evidence that microbiota are adapted to their cognate hosts further supports co‐evolutionary history with potential implication for holobiont health, although the genetic and molecular basis determining this specialization remains mostly unknown.

### Microbiota members adapt to plant immune responses

**Figure 1:**
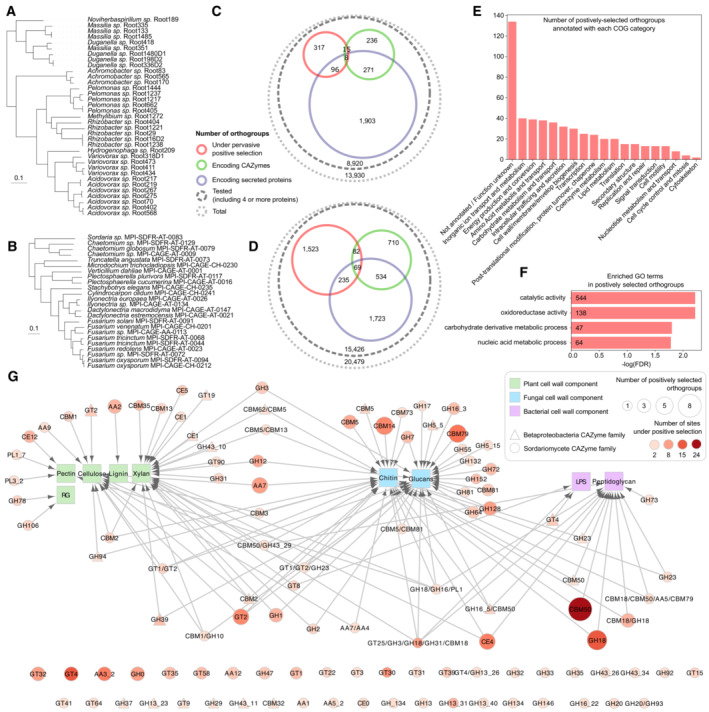
Gene families under positive selection in the *Arabidopsis thaliana* root‐associated microbiota >We performed dN/dS analyses to identify gene families under pervasive positive selection in the *A. thaliana* root microbiota (Appendix). Phylogenomic trees presenting microbiota members included in the analysis are shown on panels (A) (Betaproteobacteria) and (B) (Sordariomycetes). Counts of gene families under positive selection, encoding carbohydrate‐active enzymes (CAZymes) and encoding secreted proteins are depicted on panels (C and D), for Betaproteobacteria and Sordariomycetes respectively (Dataset EV1). Since Betaproteobacteria gene families under positive selection do not show any significant enrichment in Gene Ontology (GO) terms, panel (E) presents the number of families in each Cluster of Orthologous Groups (COG) functional category. Panel (F) shows GO terms significantly enriched in the set of Sordariomycete gene families under positive selection. On panel G, each CAZyme family under positive selection is linked to its putative substrate in plant, microbial and/or fungal cell walls (RG—Rhamnogalacturonan, LPS—Lipopolysaccharide; Dataset EV2).

Microbial adaptation to plants involves immunity avoidance and repression. The rapid evolution of genes encoding MAMPs may prevent perception by PRRs and may therefore promote microbial survival (Teixeira *et al*, 2019). Nitrogen‐fixing bacteria encode flg22 variants that do not elicit defenses in *L. japonicus*, pointing to mutations to evade immune responses (Lopez‐Gomez *et al*, 2012). Similarly, the *A. thaliana* root microbiota comprises a majority of bacteria that produce nonimmunogenic variants of flg22, shown to be under strong evolutionary pressure due to FLS2 receptor activity (Colaianni *et al*, 2021; Parys *et al*, 2021). An alternative approach to evade flg22‐triggered immunity evolved in *Pseudomonas* bacteria that repress the synthesis of flagellae (Pfeilmeier *et al*, 2016). Finally, fungi produce proteins capable of binding their MAMPs with high affinity to prevent recognition by PRRs. Typically, lysin‐motif (LysM) effectors bind cell wall chitin to prevent perception (Kombrink & Thomma, 2013). While the first LysM effector was characterized in the context of plant pathogenicity (de Jonge *et al*, 2010), such effectors are also produced by nonpathogenic fungi, such as the mycorrhizal symbiont *Rhizophagus irregularis* (Zeng *et al*, 2020). The beneficial endophyte *S. indica* furthermore produces a β‐glucan‐binding lectin, which sequesters this fungal MAMP but also induces cell wall remodeling to prevent recognition (Wawra *et al*, 2016). In root‐associated Betaproteobacteria and Sordariomycetes of *A. thaliana* (Fig 1A and B; Appendix), many genes encoding LysM proteins (CAZyme family CBM50) and other fungal cell wall‐active enzymes are under positive selection (Fig 1C–G; Datasets EV1 and EV2). While LysMs can also bind bacterial peptidoglycan and may contribute to a diversity of functions, it is likely that fungal cell wall‐binding proteins are under accelerated evolution, so fungi can avoid host detection and survive in plant microbiota. Although some microbes evolved mechanisms to avoid MAMP perception by plants, they may still elicit MTI responses. However, some commensal microbiota members evolved strategies to restrain or suppress these responses. An *A. thaliana* root‐associated community of commensal bacteria was recently shown to comprise taxonomically diverse MTI suppressor strains with efficient root colonization abilities (Teixeira *et al*, 2021). Studies of individual microbiota members revealed mechanisms behind such immunity suppression. The beneficial root‐colonizer *Pseudomonas simiae* WCS417 actively suppresses more than half of the MAMP‐triggered transcriptional responses by modulating plant auxin signaling (Stringlis *et al*, 2018b), whereas *Pseudomonas capeferrum* WCS358 produces organic acids to lower the extracellular pH, thereby suppressing the flg22‐mediated oxidative burst and transcriptional reprogramming (Yu *et al*, 2019b). In the fungal kingdom, a common MTI‐suppression strategy involves the secretion of effector proteins with diverse functions. *In planta*, expression of candidate effector genes is not limited to pathogens but is common for many fungal endophytes, suggesting that effectors are important for their successful endophytic colonization (Lahrmann *et al*, 2015; Hiruma *et al*, 2016; Mesny *et al*, 2021). Moreover, the MiSSP7 effector secreted by the mycorrhizal fungus *L. bicolor* prevents JA‐mediated transcriptional activation of immunity‐related genes, thereby promoting the ectomycorrhizal symbiosis (Plett *et al*, 2014). The phylogenetically distant arbuscular mycorrhizal fungus *R. irregularis* produces SP7 effectors that interact with the rice transcription factor ERF19 to attenuate ethylene‐mediated immune responses (Kloppholz *et al*, 2011). Similar to pathogens (Wu & Derevnina, 2023), microbiota members may have also evolved adaptive strategies to disrupt plant ETI. Some secrete proteins with domains similar in sequence to some NLR motifs, possibly interfering *in planta* with key protein interactions in the effector‐triggered immune signaling (Levy *et al*, 2017). Together, these findings show that the ability to avoid or repress immune responses constitutes an essential trait of plant‐adapted microbes.

Importantly, some microbiota members even evolved molecular mechanisms that depend on plant immune responses. While SA‐dependent defenses contribute to shaping microbiota composition, several bacterial strains benefit from SA, using it either as growth signal or as carbon source (Lebeis *et al*, 2015). A similar observation was made for root endophytic fungi of horseradish that adapted to defensive glucosinolates (Szűcs *et al*, 2018). Furthermore, oak‐adapted leaf endophytic fungi show increased tolerance when compared with generalists to host antifungal polyphenolic compounds and can even utilize some of them as carbon source (Nickerson *et al*, 2023). Root‐associated *Bacillus velezensis* bacteria are also adapted to MTI, as ROS produced during *A. thaliana* colonization stimulate bacterial auxin production, promoting efficient root colonization, but also activating plant defense against fungal pathogens (Tzipilevich *et al*, 2021). Finally, studies of individual growth‐promoting fungi and bacteria revealed that uncompromised plant immune systems are necessary for their beneficial effects. Accordingly, the fungal endophytes *S. indica* and *C. tofieldiae* are unable to promote growth of immunity‐compromised *A. thaliana* mutant lines (Lahrmann *et al*, 2015; Hiruma *et al*, 2016). Similarly, the beneficial root‐associated bacteria *Pseudomonas* sp. CH267 requires the *A. thaliana*‐specific tryptophan‐derived phytoalexin camalexin to promote plant growth (Koprivova *et al*, 2019). Thus, collectively these findings show that the ability to avoid or repress immune responses is key to persist in plant‐associated microbiota. Additionally, plant‐adapted microorganisms evolved molecular mechanisms that rely on immunity outputs.

### Efficient catabolism of plant‐derived carbohydrates underlies microbial adaptation to plants

Arguably, plant microbiota members are metabolically adapted to utilize plant‐derived carbon compounds, including cell wall components (Müller *et al*, 2016). Different plants have different cell wall composition, and cell walls of monocots and dicots are structurally clearly distinct (Vogel, 2008). Dicot cell walls are pectin‐rich (dicots: 35%, maize: 10%), whereas grass cell walls contain more hemicellulose (grasses: 60%, dicots: 30%) and phenolics (up to 5%). Major differences between the microbiota of sugarcane (monocot), *A. thaliana* (dicot), and lignin‐rich poplar trees (dicot) (Getzke *et al*, 2019) may reflect differential microbial abilities to utilize different plant cell wall components. Large‐scale comparative genomics revealed that the genomes of plant‐associated bacteria are enriched in genes involved in carbohydrate metabolism (Levy *et al*, 2017), possibly acquired from plant hosts through horizontal gene transfer (preprint: Haimlich *et al*, 2022). When investigating the functions of genes under positive selection in *A. thaliana* root‐associated Betaproteobacteria and Sordariomycetes (Fig 1A–D; Appendix; Dataset EV1), enrichment in enzyme‐encoding genes and in genes involved in the metabolism of carbohydrate derivatives was found in Sordariomycetes (Fig 1F). Since microbiota members utilize plant‐derived carbohydrates as main energy source, optimization of carbohydrate catabolism is important for microbial adaptation to plant‐associated environments. The CAZyme‐encoding gene families under positive selection in both Sordariomycetes and Betaproteobacteria comprise plant cell wall‐active enzymes acting on xylan, cellulose, and pectin (Fig 1G; Dataset EV2), the main constituents of *A. thaliana* cell walls (Bacic, 2006). Moreover, a positively selected fungal pectin‐degrading enzyme constitutes a key genetic determinant underlying efficient colonization of the *A. thaliana* root endosphere (Mesny *et al*, 2021). Cellulose catabolism has also been suggested to rule microbiota assembly, as genetic modification of rice plants revealed that accumulation of cellulose in leaves caused a shift in community composition (preprint: Roman‐Reyna *et al*, 2019). Interestingly, the evolution of arbuscular mycorrhizal symbiosis correlated with substantial losses of plant cell wall‐degrading enzymes (Malar C *et al*, 2021), and fungal nutrition relies on plant‐encoded molecular mechanisms, as plant invertases convert sucrose into glucose and fructose before transfer to the fungal symbiont via monosaccharide transporters (Salvioli di Fossalunga & Novero, 2019). This points to a co‐evolutionary model where plants modulate carbon allocation to “reward,” and thus select, the symbiont (Kiers *et al*, 2011). Taken together, these findings suggest that evolution of plant cell wall‐degradation ability, and more generally carbohydrate metabolism, likely drives plant‐microbiota co‐evolution.

### Rapid microbial adaptation may result in mutualism

Experimental evolution suggests that microbes may rapidly evolve towards mutualism *in planta*. Detrimental *Pseudomonas protegens* evolved beneficially in the rhizosphere of *A. thaliana* within six plant growth cycles (Li *et al*, 2021). Interestingly, after co‐cultivating the yeast *Saccharomyces cerevisiae* with the alga *Chlamydomonas reinhardtii*, many yeast adaptive mutations were identified, ranging from competitive to mutualistic (Venkataram *et al*, 2023). While the presence of the alga did not determine which mutations were adaptive, it favored yeast mutants promoting both fungal and algal yields, resulting in stronger mutualism. To decipher the emergence of root nodule symbioses, pathogenic *Ralstonia solanacearum* bacteria transformed with rhizobial nitrogen‐fixation and nodulation genes were inoculated onto *Mimosa pudica* legume plants (Marchetti *et al*, 2010). While this did not induce nodule formation upon bacterial infection, rapid transition to mutualism occasionally occurred due to adaptive mutations that inactivated type‐III secretion. Finally, after repeated inoculations onto five *Medicago truncatula* accessions, the nitrogen‐fixing bacteria *Ensifer meliloti* rapidly became more beneficial toward the host (Batstone *et al*, 2020), confirming that microbial adaptation can be highly host‐specific (Schirrmann & Leuchtmann, 2015; Wippel *et al*, 2021). Thus, microorganisms can evolve into mutualistic symbionts over short evolutionary time frames, demonstrating their capacity to rapidly adapt to plant‐associated environments, with implications for host health. By evolving plant‐beneficial properties, microbiota members can sustain the health of a host, which likely supports their fitness in turn.

To persist in nature, microbes must also evolve ways to survive upon host death. A recent study of the interaction between *Sulfitobacter* D7 and microalga *Emiliania huxleyi* revealed that by sensing algal metabolites, *Sulfitobacter* can switch from coexistence to pathogenicity by upregulating flagellar motility and diverse transport systems, presumably to maximize assimilation of metabolites released upon algal cell death (Noa Barak‐Gavish *et al*, 2023). Also, some fungal microbiota members switch lifestyle to persist in soil after host death. Most oak‐associated leaf endophytes show saprophytic capacity to survive in soil litter (Davis *et al*, 2023). In *Serendipita indica*, transition to a saprotrophic lifestyle is inhibited by host signals (Lahrmann *et al*, 2013). Mutualism can therefore rapidly arise from plant‐microbe co‐evolution, but microbial fitness is also dependent on the ability to switch lifestyle and survive upon environmental changes.

## Intermicrobial competition evolved to protect the holobiont

While plant cell walls and immune responses constitute major forces driving microbial evolution in holobionts, microbiota members are connected in complex networks of positive and negative interactions. We will first show that plant microbiota are shaped by these interactions, particularly by highly interactive keystone microbes. We will then present how microbiota are structured by intermetabolic dependencies and by microbial antagonism. Finally, we argue that antagonistic intermicrobial interactions can protect holobionts against disease.

### Keystone microbes shape microbiota structures

As for any microbial community, interactions between microorganisms structure plant microbiota and drive their assemblies (Hunter *et al*, 2010; Kemen, 2014; Hassani *et al*, 2018). In theory, two microorganisms in each community can interact positively, negatively, or not at all. Microbes showing the highest number of interactions are referred to as “hubs” and are thought to be keystones of community structures. If hub microbial presence is affected, major community shifts are typically observed. For instance, in the *A. thaliana* phyllosphere, environmental conditions and host genotypic factors directly impact the presence of two eukaryotic hub microbes, the oomycete *Albugo laibachii* and the fungus *Dioszegia* sp., with cascading consequences on bacterial colonization capabilities and community composition (Agler *et al*, 2016). Fungi generally represent hubs in plant microbiota, influencing bacterial community composition (Hassani *et al*, 2018). Mycorrhizal fungi influence bacterial assemblages on grass roots (Singh *et al*, 2008), and distinct arbuscular mycorrhizal fungi recruit different bacterial communities to the same plant species (Zhou *et al*, 2020). Importantly, the presence and identity of nonfungal symbionts, especially nitrogen‐fixing bacteria, impact root‐associated microbial communities (Uroz *et al*, 2019). These findings prove that intermicrobial interactions structure plant‐associated communities and demonstrate the importance of hub microbes for microbiota assemblies.

### Metabolic interdependencies shape microbiota composition

**Figure 2:**
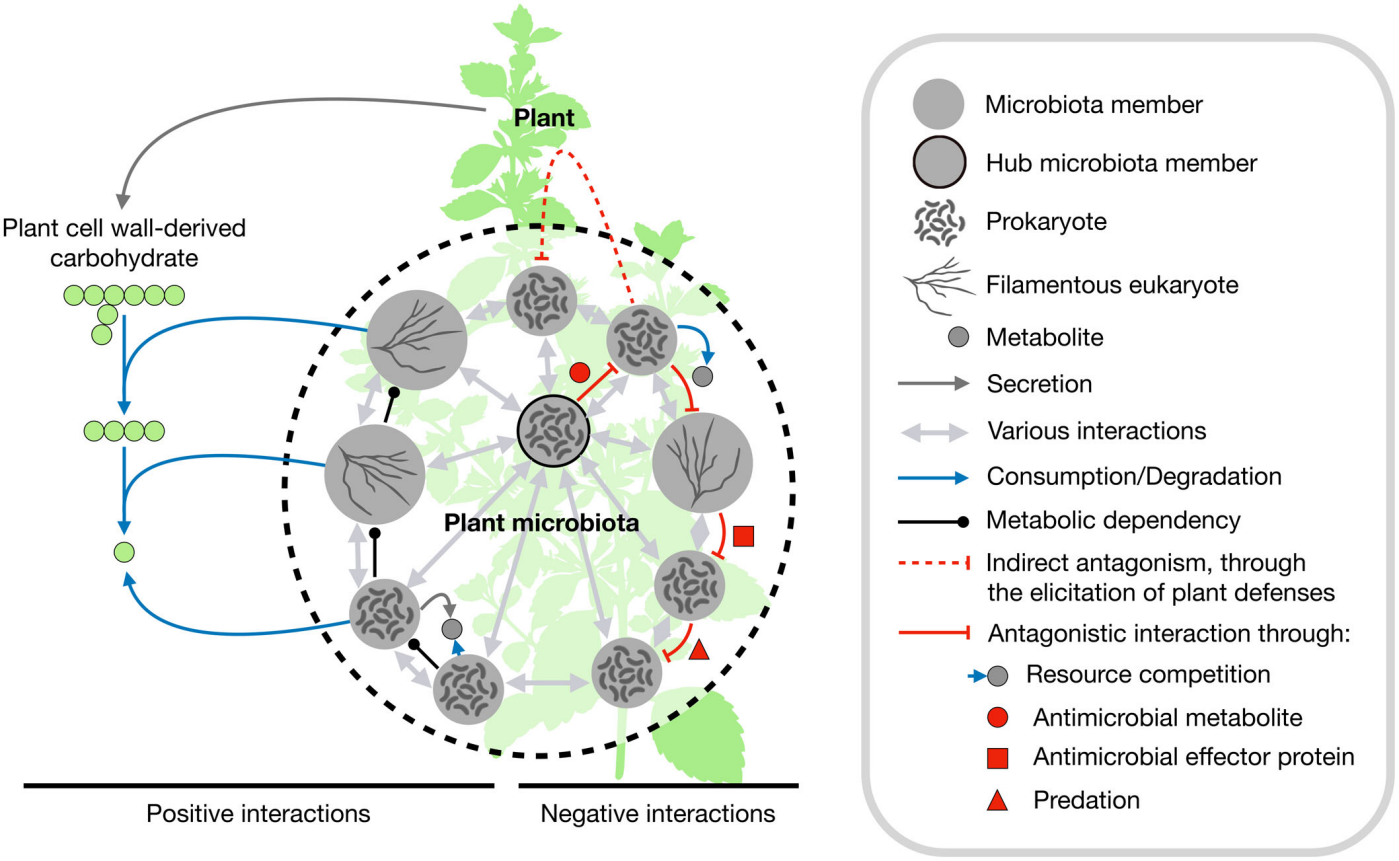
Intermicrobe interactions drive community structure (Left) Positive interactions between microbiota members mostly consist in cross‐feeding and can be observed as organized hierarchically. (Right) Antagonism between microbiota members can be either direct or indirect and relies on a diversity of mechanisms.

While interaction processes favoring microbial coexistence within plant holobionts remain poorly understood (Hassani *et al*, 2018), metabolic interdependencies have been described in synthetic microbial communities (Pontrelli *et al*, 2022). When artificially feeding a community with a single carbohydrate polymer (e.g., a plant cell wall component), it is possible to distinguish degraders that digest the polymer to acquire nutrients from exploiters that feed on the resulting digestion products and are therefore dependent on the presence of the initial degraders, and from scavengers that rely exclusively on metabolites produced by degraders and exploiters and thus depend on their presence for growth (Fig 2). Importantly, microbes can change between trophic levels during their life cycle and scavengers eventually implement positive feedback loops supporting the growth of exploiters and degraders that provide their preferred by‐products (Sulheim & Mitri, 2023). However, such simplified tri‐trophic hierarchical community structure illustrates that microbial interdependency consists essentially of cross‐feeding, generally termed “syntrophy,” and relies on different metabolic capabilities. A single limiting carbon compound was shown to rule the assembly of highly diverse communities of leaf‐isolated bacteria *in vitro* (Goldford *et al*, 2018; Murillo‐Roos *et al*, 2022). These communities subsist through cross‐feeding owing to differential metabolic capabilities. Bacteria without the ability to utilize the available carbon source can survive by utilizing only secreted or leaked metabolites from other community members (Murillo‐Roos *et al*, 2022). Arguably, metabolic interdependencies evolve through resource‐saving gene losses, occurring when the synthesis of a compound becomes dispensable, as provided by community co‐inhabitants (Morris *et al*, 2012). Importantly, by nutritional selective pressure on bacterial interactions, plants shape intermetabolic dependencies (Mataigne *et al*, 2022). Evolutionary selection will favor the assembled community if beneficial for the plant. In turn, bacterial production of specific metabolites will be selected if cross‐feeding results in a reward for the producer, for example, through nutrients from the plant.

Since the ability to degrade plant cell wall components is one of their key traits, fungi act as essential degraders in plant microbiota, further affirming their hub status (Nagy *et al*, 2017). While feeding on complex cell wall components, fungal microbiota members produce metabolites that maintain communities of bacteria along their hyphae, referred to as the “hyphosphere” (Wang *et al*, 2022). For instance, arbuscular mycorrhizal fungi carry bacteria along their extraradical hyphae that are fed by hyphal exudates and enhance mineralization of organic phosphorus (Jiang *et al*, 2021). To reach the legume rhizosphere and trigger nodulation, rhizobia use mycelia as dispersal networks, likely benefiting from fungal exudates (Zhang *et al*, 2020). In fact, diverse soil bacteria were shown to form biofilms on the hyphae of ectomycorrhizal fungi that are modulated by plant‐ and fungi‐encoded mechanisms (Guennoc *et al*, 2018). Thus, metabolic interdependencies constitute key forces underlying microbiota assembly.

### Intermicrobial competition and antagonism shape microbiota composition

While microbes coexist in communities through cross‐feeding and benefit from each other, negative interactions similarly shape microbiota assemblies (Fig 2). A survey of antagonistic interactions among rhizosphere soil, root, and leaf bacteria of the medicinal plant *Echinacea purpurea* revealed that microbial antagonistic abilities and sensitivities differ based on taxonomy, but also ecological niche (Maida *et al*, 2016). Bacteria isolated from aboveground tissues were much more sensitive to antagonism than bacteria from underground compartments. A high‐throughput screening of binary interactions among 224 bacteria isolated from *A. thaliana* leaves linked antagonistic activities to biosynthetic gene clusters and showed that *Brevibacillus* sp. is a potent antagonist of microbiota co‐inhabitants through the synthesis of antimicrobials such as streptocidins, phosphobrevin, macrobrevin, and marthiapeptide (Helfrich *et al*, 2018). A similar analysis of binary interactions among 198 *A. thaliana* root‐isolated bacteria identified *Pseudomonas brassicacearum* as a potent antagonist that relies on the combined action of two exometabolites, an antimicrobial and an iron chelator, that suppress competitors, and thereby promote its root colonization (Getzke *et al*, 2023).

Resource competition represents an important mechanism for indirect microbial antagonism (Hassani *et al*, 2018). The ability to rapidly utilize a limited resource can be detrimental for a less competitive microbe. Additionally, microbes can sequester resources, preventing utilization by other community members. Siderophores chelate soil iron for microbial uptake and are differentially exploitable to prevent other microbes to obtain essential iron resources (Joshi *et al*, 2006). For instance, pyoverdine is an iron‐chelator produced by some *A. thaliana* root‐associated bacteria contributing to their competitiveness (Getzke *et al*, 2023).

Microorganisms also directly suppress growth of microbial opponents. Some *Pseudomonas* bacteria produce 2,4‐diacetylphloroglucinol, a secondary metabolite with both antibacterial and antifungal properties, affecting root‐associated bacteria and fungi of *A. thaliana* and wheat (Bakker *et al*, 2002; Getzke *et al*, 2023). Additionally, antimicrobial volatile organic compounds may be produced, for instance by potato‐isolated *Pseudomonas* strains to antagonize oomycete growth (De Vrieze *et al*, 2015). Endophytic fungi are well‐known to produce antibiotic metabolites (Martinez‐Klimova *et al*, 2017). For instance, *Fusarium* and *Alternaria* spp., frequently found in *A. thaliana* root microbiota (Thiergart *et al*, 2020), secrete metabolites that restrain the growth of other microbes (Martinez‐Klimova *et al*, 2017).

Intriguingly, it was recently uncovered that fungi exploit effectors with antibacterial and antifungal activities that modulate the composition of plant‐associated communities (Snelders *et al*, 2020, 2021, 2023). This extends the range of previously considered effector activities, typically thought‐out to mainly act in manipulation of host physiology to support host colonization, frequently through modulation of host immune signaling. While effectors with antimicrobial activity were initially described in the context of plant infection by the pathogenic soil‐borne and broad host‐range pathogen *Verticillium dahliae*, other fungal pathogens may produce such effectors too. More recently, it was reported that the soil‐borne white root rot pathogen *Rosellinia necatrix* expresses antimicrobial effector proteins during host colonization, while smut fungi express a conserved extracellular ribonuclease with broad‐spectrum cytotoxic activity to compete with host‐associated bacteria on the leaves of host plants (preprint: Chavarro‐Carrero *et al*, 2023; preprint: Ökmen *et al*, 2023). Pathogenic oomycetes seem to rely on a similar strategy, since *Albugo candida* secretes antimicrobials into the apoplast of *A. thaliana* leaves to repress the growth of keystone bacteria (Gómez‐Pérez *et al*, 2023). The expected occurrence of these effectors across the fungal kingdom, and beyond, suggests that such effectors are likely produced by fungi with diverse lifestyles, including commensal fungi in plant microbiota (Snelders *et al*, 2022). Although several such effectors may result from inter‐microbial co‐evolution (Snelders *et al*, 2022), antimicrobial effectors of plant‐associated filamentous eukaryotes broadly evolved to support fungal accommodation in the holobiont, since some are multifunctional and target plant processes.

Finally, a third intermicrobial antagonism mechanism that contributes to plant microbiota assembly is predation. Mycophagous bacteria that grow at the expense of living fungal hyphae occur in plant microbiota (Rudnick *et al*, 2015; Hassani *et al*, 2018). Furthermore, the bacterial prey range of the predator *Bdellovibrio* spp. isolated from roots of common bean suggests adaptation to this environment, as most targets are root‐associated bacteria (Jurkevitch *et al*, 2000).

### Plant microbiota mediate disease suppression

By hosting antagonistic microorganisms, plants extend their capacity to prevent disease development. This microbiota function is essential for host survival and persistence, and thus a driving force for plant‐microbiota co‐evolution. The “cry for help” theory proposes exudate‐mediated recruitment of disease‐suppressive microbiota by plants facing pathogen attack, but it remains to be validated (Rizaludin *et al*, 2021). Roots of *A. thaliana* naturally host fungi with high pathogenic potential, especially *Fusarium*, *Plectosphaerella* and *Ilyonectria* (Mesny *et al*, 2021). Detrimental overcolonization by these fungi is prevented by bacterial root commensals, including Pseudomonadaceae and Comamonadaceae, that display particularly high antifungal activities in binary interaction assays (Durán *et al*, 2018). In the *A. thaliana* phyllosphere, the oomycete hub *Albugo laibachii* affects plant health unless controlled by a lysozyme secreted by the yeast *Moesziomyces bullatus* (Eitzen *et al*, 2021). Similarly, the bentgrass leaf endophyte *Epichloë festucae* secretes an antifungal protein that antagonizes the dollar spot pathogen *Clarireedia jacksonii* (Fardella *et al*, 2022). Upon sugar beet infection by *Rhizoctonia solani*, root endophytic bacteria prevent disease development by production of antifungal secondary metabolites (Carrión *et al*, 2019). Protective microbiota members also rely on the emission of volatile organic compounds to antagonize pathogens. Maize rhizosphere *Pseudomonas* release volatiles that significantly affect mycelial growth of *Fusarium proliferatum* (Cordero *et al*, 2014). Plant protection can additionally occur via production of microbial physical barriers, as endophytic *Enterobacter* form biofilm‐mediated microcolonies and assemble multilayer root‐hair endophyte stacks to constitute a physical barrier that traps *Fusarium graminearum* (Mousa *et al*, 2016). Tomato root endophytic *Rahnella aquatilis* bacteria counteract *F. oxysporum*‐induced rhizosphere alkalinization through secretion of gluconic acid while moving through hyphae to reach and colonize plant roots, thereby preventing fungal infection (Palmieri *et al*, 2020). Interestingly, the poplar phyllosphere includes diverse fungal endophytes that increase or decrease *Melampsora* leaf rust disease (Busby *et al*, 2016). In a community context, the combined effect of individual microbial antagonistic activities can result in plant protection. Consistently, synergistically with root‐associated bacteria the fungal root endophyte *Serendipita vermifera* protects plants against the soil‐borne fungal pathogen *Bipolaris sorokiniana* (Mahdi *et al*, 2021). Finally, rhizosphere bacteria of *Mikania micrantha* preventing harm from soil‐resident *Fusarium* fungi and *Ralstonia* bacteria were associated with plant invasiveness, demonstrating the contribution of disease‐protective microbiota members to fitness (Yin *et al*, 2020). Therefore, plant protection from pathogens represents a key function of the microbiota, mostly through antagonistic capabilities. Similar to plant‐microbe co‐evolution, evolutionary arms races likely rule microbial evolution within holobionts. As previously mentioned, the wilt pathogen *V. dahliae* evolved antimicrobial effector proteins targeting antagonists in the plant microbiota to successfully colonize the plant and cause disease (Snelders *et al*, 2020, 2021, 2022, 2023). Under the selection pressure of the antimicrobials secreted by *V. dahliae*, protective strains insensitive to these effectors will be selected. Consistent with this hypothesis, plant‐pathogenic *R. solanacearum* adapts to growth‐inhibiting *Bacillus amyloliquefaciens* VOCs, although this increased tolerance negatively affected *R. solanacearum* virulence (Wang *et al*, 2023).

Importantly, disease suppression also occurs through microbiota‐induced elicitation of plant immunity (Fig 2). The physiological state of enhanced immunity induced by microbiota members is commonly referred to as “induced systemic resistance” (Trivedi *et al*, 2020). For instance, the protective ability of leaf‐isolated bacteria against the bacterial pathogen *Pseudomonas syringae* pv. tomato is reduced in MTI‐compromised *A. thaliana* (Vogel *et al*, 2021). Axenic *A. thaliana* are hypersusceptible to this foliar pathogen, whereas plants recolonized with synthetic and soil‐derived communities show substantially restored immunocompetence (preprint: Paasch *et al*, 2023). Further studies are needed to better characterize the relative contributions of intermicrobial antagonism and induced systemic resistance in disease suppression.

## Conclusion and outlook

**Figure 3:**
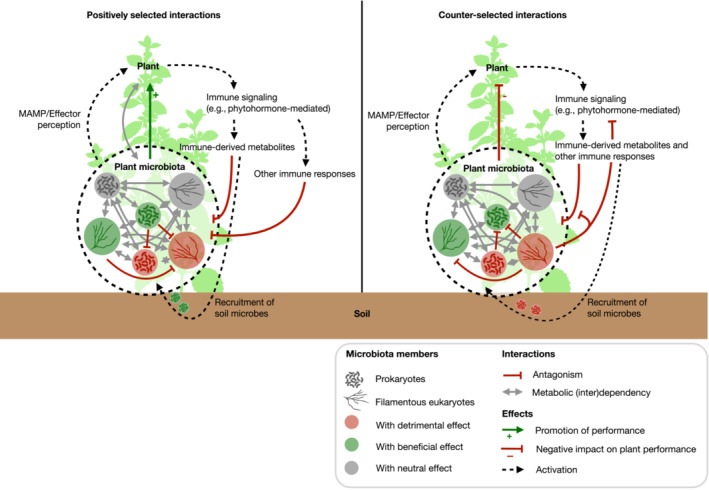
Positively selected interactions within the holobiont ultimately promote plant performance (Left) Graphical summary of the interactions reviewed in this article, that are positively selected by the plant in the holobiont and together, ultimately contribute to promoting plant performance. (Right) Graphical representation of interactions that are likely counter‐selected for through plant‐mediated mechanisms, according to the holobiont hypothesis, since they would ultimately result in negative impact on plant performance.

Complex plant–microbe and intermicrobial interactions in plant holobionts can result in beneficial outcomes for the plant (Fig 3). We argue that most of these interactions evolved within plant holobionts and have been selected to maintain an environment promoting the survival and fitness of the whole holobiont. On one side, plants evolved mechanisms that shape their microbiota, such as immune responses and secreted metabolites. This arsenal is key to protect plants from disease, as it fights pathogens and prevents dysbiosis. Moreover, plants evolved the capacity to actively recruit beneficial microorganisms that contribute to their performance and their resistance to biotic and abiotic stresses. On the microbial side, genomes of microbiota members carry signatures of adaptation to plant‐associated environments, resulting in gene repertoire modifications for carbohydrate metabolism and allowing efficient utilization of plant carbon compounds. Recent findings based on experimental evolution suggest that mutualism can rapidly arise from evolution of microorganisms in close association with plant hosts. Importantly, plant‐adapted microbes overcome plant immune outputs, and sometimes benefit from them. Therefore, microbial adaptation results in an improved capacity to persist in plant‐associated environments. Finally, within plant microbiota, microorganisms are tightly interconnected, through metabolic interdependencies and antagonistic relationships. While metabolic interdependencies rule microbiota assemblies, in combination with the plant immune system, antagonism mitigates detrimental activities of microbiota co‐inhabitants with high pathogenic potential. This microbial defense layer contributes to disease protection, and thereby supports holobiont persistence. Thus, because of their direct impact on plant performance, interactions within holobionts are under strong selection pressure. However, their evolutionary dynamics remain poorly understood (Box 3).

Box 3 **In need of answers**

- How are holobionts shaped, and are plant‐microbe interactions more important drivers than inter‐microbial ones? Are the co‐evolutionary dynamics of a given plant‐microbe association affected by the presence of microbiota co‐inhabitants?
- Do holobiont subcommunities exhibit different dynamics? How persistent are selected interactions across time and upon environmental changes?
- What plant factors govern holobiont composition? Can we confirm the key structuring roles of plant cell wall composition and exudates? Besides MTI pathways, do NLR catalogs play a determining role?
- How convergent is the evolution of phylogenetically distant microbes that adapt to the same host? Is a core set of microbial functions required?
- Is the invasiveness of a microbe correlated with its pathogenic potential? To what extent have microbes with a high pathogenic potential evolved to manipulate the microbiota of the plant holobiont in order to colonize the host?

## Author contributions

**Fantin Mesny:** Conceptualization; funding acquisition; writing – original draft; writing – review and editing. **Stéphane Hacquard:** Supervision; funding acquisition; writing – review and editing. **Bart PHJ Thomma:** Conceptualization; supervision; funding acquisition; writing – review and editing.

## Disclosure and competing interests statement

The authors declare that they have no conflict of interest.

## Supporting information



Appendix

Dataset EV1

Dataset EV2
