# Supplementary material for: Co‐evolution within the plant holobiont drives host performance
Source: EMBO Rep. 2023 Jul 20;24(9):e57455. doi: 10.15252/embr.202357455 (PMC10481671; doi:10.15252/embr.202357455)
Supplement: Supplementary file 1 — Appendix [file EMBR-24-e57455-s002.pdf]

Appendix for “Co-evolution within the plant holobiont drives host performance”

<u>Table of contents:</u>

Figure 1 Appendix Methods……………………………………………………………….2

Figure 1 Appendix Methods

Analysis of gene families under positive selection in the *Arabidopsis thaliana* root-associated microbiota

We carried out a dN/dS analysis to identify genes under positive selection in the *A. thaliana* root microbiota. Such analysis requires to compare orthologous gene sequences over a set of phylogenetically related organisms. Therefore, we performed two independent analyses on Betaproteobacteria and Sordariomycetes, *i.e.* the most abundant bacterial and fungal phylogenetic classes in European *A. thaliana* root microbiota (Thiergart *et al*, 2020). We used previously published genomes of 35 Betaproteobacteria (Bai *et al*, 2015) and 24 Sordariomycete fungi (Mesny *et al*, 2021) isolated from the roots of healthy *A. thaliana* plants. CDS and amino acid sequences associated to predicted genes in genome assemblies were downloaded in January 2023 from the AtSphere website (https://www.at-sphere.com) and from the Mycocosm portal (https://mycocosm.jgi.doe.gov; Grigoriev *et al*, 2014), for bacteria and fungi respectively. Genes encoding secreted proteins were identified using predictor SignalP v6.0 (Teufel *et al*, 2022). Carbohydrate-active enzymes (CAZymes) were annotated using dbCan v2 (Zhang *et al*, 2018). Annotation software emapper v2.0 (relying on database EggNog v5) was used to annotate bacterial genes with Gene Ontology (GO) terms and Clusters of Orthologous Groups (COG) categories (Cantalapiedra *et al*, 2021). Fungal gene annotations in GO terms were downloaded from the Mycocosm portal.

To classify proteins into orthogroups, we conducted two independent orthology prediction analyses (OrthoFinder v2.5.4; Emms & Kelly, 2019). The OrthoFinder software implements the phylogenetic tree reconstruction method STAG (Emms & Kelly, 2018) that estimates species trees from individual orthogroup phylogenetic trees (Betaproteobacteria: **Fig 1A;** Sordariomycetes: **Fig 1B**). To identify gene families under pervasive positive selection, we conducted dN/dS analyses on all orthogroups of minimum four proteins. First, protein sequences in each orthogroup were aligned with FAMSA v1.6.1 (Deorowicz *et al*, 2016). Then, codon alignments were obtained using PAL2NAL v14 (Suyama *et al*, 2006). Finally, we used the algorithm HyPhy FUBAR v2.2 (Murrell *et al*, 2013) to obtain Bayesian predictions of positively selected codons. If minimum two codons were predicted as positively selected, the gene family was categorized as positively selected (**Dataset EV1**).

To characterize gene functions under selection in the *A. thaliana* root microbiota, we observed overlaps between gene families under positive selection and families encoding secreted proteins and/or CAZymes (Betaproteobacteria: **Fig 1C**; Sordariomycetes: **Fig 1D**). Fisher's exact tests revealed that orthogroups under positive selection are not significantly enriched for secreted protein- and CAZyme-encoding genes (p>0.05). We performed GO term enrichment analyses using GOATOOLS v1.1.5 (Klopfenstein *et al*, 2018) and gene annotations in GO terms. Since no significant enrichment was identified for Betaproteobacteria, we analyzed the number of positively selected orthogroups annotated in each COG category (**Fig 1E**). Significantly enriched GO terms in orthogroups under positive selection for Sordariomycetes are shown in **Fig 1F**. Since carbohydrate-related functions are enriched in Sordariomycete gene families under positive selection, we further analyzed positively selected CAZyme-encoding genes. Each bacterial and fungal CAZyme family that is under positive selection was linked to its known possible substrates in plant, fungal or bacterial cell walls, as documented on website http://www.cazy.org/ (visited in January 2023, Drula *et al*, 2022; **Dataset EV2**). A network representation was produced using Cytoscape v3.7.2 (Shannon *et al*, 2003) and is shown on **Fig 1G**.

To ensure reproducibility and further document this analysis, genomic data and scripts have been deposited on GitHub: https://github.com/fantin-mesny/Scripts-from-review-Mesny-et-al.-2023-.

References

Bai Y, Müller DB, Srinivas G, Garrido-Oter R, Potthoff E, Rott M, Dombrowski N, Münch PC, Spaepen S, Remus-Emsermann M et al (2015) Functional overlap of the *Arabidopsis* leaf and root microbiota. *Nature* 528: 364–369

Cantalapiedra CP, Hernández-Plaza A, Letunic I, Bork P, Huerta-Cepas J (2021) eggNOG-mapper v2: functional annotation, orthology assignments, and domain prediction at the metagenomic scale. *Mol Biol Evol* 38: 5825–5829

Deorowicz S, Debudaj-Grabysz A, Gudys A (2016) FAMSA: fast and accurate multiple sequence alignment of huge protein families. *Sci Rep* 6: 1–13

Drula E, Garron M-L, Dogan S, Lombard V, Henrissat B, Terrapon N (2022) The carbohydrate-active enzyme database: functions and literature. *Nucleic Acids Res* 50: D571–D577

Emms DM, Kelly S (2018) STAG: Species tree inference from all genes. *bioRxiv* https://doi.org/10.1101/267914 [PREPRINT]

Emms DM, Kelly S (2019) OrthoFinder: phylogenetic orthology inference for comparative genomics. *Genome Biol* 20: 1–14

Grigoriev IV, Nikitin R, Haridas S, Kuo A, Ohm R, Otillar R, Riley R, Salamov A, Zhao X, Korzeniewski F *et al* (2014) MycoCosm portal: gearing up for 1000 fungal genomes. *Nucleic Acids Res* 42: D699–D704

Klopfenstein DV, Zhang L, Pedersen BS, Ramírez F, Vesztrocy AW, Naldi A, Mungall CJ, Yunes JM, Botvinnik O, Weigel M *et al* (2018) GOATOOLS: a Python library for gene ontology analyses. *Sci Rep* 8: 1–17

Mesny F, Miyauchi S, Thiergart T, Pickel B, Atanasova L, Karlsson M, Hüttel B, Barry KW, Haridas S, Chen C *et al* (2021) Genetic determinants of endophytism in the *Arabidopsis* root mycobiome. *Nat Commun* 12: 1–15

Murrell B, Moola S, Mabona A, Weighill T, Sheward D, Kosakovsky Pond SL, Scheffler K (2013) FUBAR: a fast, unconstrained bayesian approximation for inferring selection. *Mol Biol Evol* 30: 1196–1205

Shannon P, Markiel A, Ozier O, Baliga NS, Wang JT, Ramage D, Amin N, Schwikowski B, Ideker T (2003) Cytoscape: a software environment for integrated models of biomolecular interaction networks. *Genome Res* 13: 2498–2504

Suyama M, Torrents D, Bork P (2006) PAL2NAL: robust conversion of protein sequence alignments into the corresponding codon alignments. *Nucleic Acids Res* 34: W609–W612

Teufel F, Almagro Armenteros JJ, Johansen AR, Gíslason MH, Pihl SI, Tsirigos KD, Winther O, Brunak S, von Heijne G, Nielsen H (2022) SignalP 6.0 predicts all five types of signal peptides using protein language models. *Nat Biotechnol* 40: 1023–1025

Thiergart T, Durán P, Ellis T, Vannier N, Garrido-Oter R, Kemen E, Roux F, Alonso-Blanco C, Ågren J, Schulze-Lefert P *et al* (2020) Root microbiota assembly and adaptive differentiation among European *Arabidopsis* populations. *Nat Ecol Evol* 4: 122–131

Zhang H, Yohe T, Huang L, Entwistle S, Wu P, Yang Z, Busk PK, Xu Y, Yin Y (2018) dbCAN2: a meta server for automated carbohydrate-active enzyme annotation. *Nucleic Acids Res* 46: W95–W101
